# Supplementary material for: Gell: A GPU-powered 3D hybrid simulator for large-scale multicellular system
Source: PLoS One. 2023 Jul 18;18(7):e0288721. doi: 10.1371/journal.pone.0288721 (PMC10353785; doi:10.1371/journal.pone.0288721)
Supplement: Attachment — Submitted filename: Gell_Response to Reviewers.docx [file pone.0288721.s001.docx]

Dear Editor and Reviewers,

Thank you for your review and comments that have helped us improve the manuscript. We have responded to individual comments and questions as follows and made corresponding changes in the manuscript. We look forward to making any additional improvements if necessary.

Best,

Authors

PONE-D-23-02925

Gell: A GPU-powered 3D hybrid simulator for large-scale multicellular system

PLOS ONE

Dear Dr. Sheng,

Thank you for submitting your manuscript to PLOS ONE. After careful consideration, we feel that it has merit but does not fully meet PLOS ONE’s publication criteria as it currently stands. Therefore, we invite you to submit a revised version of the manuscript that addresses the points raised during the review process.

The reviewers' comments highlighted that the manuscript is well-written and the results achieved are promising. However, there are a few concerns that the authors must address. A better description of the proposed parallelization is required, including GPU settings and library versions. A comparison between the accuracy of the proposed approach and state-of-art strategies is needed. Please refer to all the reviewers' reports for detailed comments which can help improve the current version of the manuscript

Please submit your revised manuscript by Jun 16 2023 11:59PM. If you will need more time than this to complete your revisions, please reply to this message or contact the journal office at [plosone@plos.org](mailto:plosone@plos.org). When you're ready to submit your revision, log on to [https://www.editorialmanager.com/pone/](https://urldefense.com/v3/__https:/www.editorialmanager.com/pone/__;!!LQC6Cpwp!rFVHO60ZNWeVXmKrYxR6pcOSVvXfm9Xixl7jUFVMroxX7drY1cdUKYzx3AzcnTfnP9xrQFDrb0GMgDfGTw$) and select the 'Submissions Needing Revision' folder to locate your manuscript file.

Please include the following items when submitting your revised manuscript:

1. A rebuttal letter that responds to each point raised by the academic editor and reviewer(s). You should upload this letter as a separate file labeled 'Response to Reviewers'.
2. A marked-up copy of your manuscript that highlights changes made to the original version. You should upload this as a separate file labeled 'Revised Manuscript with Track Changes'.
3. An unmarked version of your revised paper without tracked changes. You should upload this as a separate file labeled 'Manuscript'.

If you would like to make changes to your financial disclosure, please include your updated statement in your cover letter. Guidelines for resubmitting your figure files are available below the reviewer comments at the end of this letter.

If applicable, we recommend that you deposit your laboratory protocols in protocols.io to enhance the reproducibility of your results. Protocols.io assigns your protocol its own identifier (DOI) so that it can be cited independently in the future. For instructions see: [https://journals.plos.org/plosone/s/submission-guidelines#loc-laboratory-protocols](https://urldefense.com/v3/__https:/journals.plos.org/plosone/s/submission-guidelines*loc-laboratory-protocols__;Iw!!LQC6Cpwp!rFVHO60ZNWeVXmKrYxR6pcOSVvXfm9Xixl7jUFVMroxX7drY1cdUKYzx3AzcnTfnP9xrQFDrb0HyXzR4wQ$). Additionally, PLOS ONE offers an option for publishing peer-reviewed Lab Protocol articles, which describe protocols hosted on protocols.io. Read more information on sharing protocols at [https://plos.org/protocols?utm_medium=editorial-email&utm_source=authorletters&utm_campaign=protocols](https://urldefense.com/v3/__https:/plos.org/protocols?utm_medium=editorial-email&utm_source=authorletters&utm_campaign=protocols__;!!LQC6Cpwp!rFVHO60ZNWeVXmKrYxR6pcOSVvXfm9Xixl7jUFVMroxX7drY1cdUKYzx3AzcnTfnP9xrQFDrb0Fn1t4dVg$).

We look forward to receiving your revised manuscript.

Kind regards,

Andrea Tangherloni
Academic Editor
PLOS ONE

Journal Requirements:

When submitting your revision, we need you to address these additional requirements.

1. Please ensure that your manuscript meets PLOS ONE's style requirements, including those for file naming. The PLOS ONE style templates can be found at 
[https://journals.plos.org/plosone/s/file?id=wjVg/PLOSOne_formatting_sample_main_body.pdf](https://urldefense.com/v3/__https:/journals.plos.org/plosone/s/file?id=wjVg*PLOSOne_formatting_sample_main_body.pdf__;Lw!!LQC6Cpwp!rFVHO60ZNWeVXmKrYxR6pcOSVvXfm9Xixl7jUFVMroxX7drY1cdUKYzx3AzcnTfnP9xrQFDrb0HccI55Cw$) and 
[https://journals.plos.org/plosone/s/file?id=ba62/PLOSOne_formatting_sample_title_authors_affiliations.pdf](https://urldefense.com/v3/__https:/journals.plos.org/plosone/s/file?id=ba62*PLOSOne_formatting_sample_title_authors_affiliations.pdf__;Lw!!LQC6Cpwp!rFVHO60ZNWeVXmKrYxR6pcOSVvXfm9Xixl7jUFVMroxX7drY1cdUKYzx3AzcnTfnP9xrQFDrb0GvgzIt6Q$)

Tiff figure files are prepared.

2. We note that the grant information you provided in the ‘Funding Information’ and ‘Financial Disclosure’ sections do not match.

When you resubmit, please ensure that you provide the correct grant numbers for the awards you received for your study in the ‘Funding Information’ section.

We corrected the error.

3. In your Data Availability statement, you have not specified where the minimal data set underlying the results described in your manuscript can be found. PLOS defines a study's minimal data set as the underlying data used to reach the conclusions drawn in the manuscript and any additional data required to replicate the reported study findings in their entirety. All PLOS journals require that the minimal data set be made fully available. For more information about our data policy, please see [http://journals.plos.org/plosone/s/data-availability](https://urldefense.com/v3/__http:/journals.plos.org/plosone/s/data-availability__;!!LQC6Cpwp!rFVHO60ZNWeVXmKrYxR6pcOSVvXfm9Xixl7jUFVMroxX7drY1cdUKYzx3AzcnTfnP9xrQFDrb0GznOG3hA$).

Generated HDS simulation results have been uploaded to the github repository.

<https://github.com/PhantomOtter/Gell/tree/main/data>

"Upon re-submitting your revised manuscript, please upload your study’s minimal underlying data set as either Supporting Information files or to a stable, public repository and include the relevant URLs, DOIs, or accession numbers within your revised cover letter. For a list of acceptable repositories, please see [http://journals.plos.org/plosone/s/data-availability#loc-recommended-repositories](https://urldefense.com/v3/__http:/journals.plos.org/plosone/s/data-availability*loc-recommended-repositories__;Iw!!LQC6Cpwp!rFVHO60ZNWeVXmKrYxR6pcOSVvXfm9Xixl7jUFVMroxX7drY1cdUKYzx3AzcnTfnP9xrQFDrb0GLTuQ60A$). Any potentially identifying patient information must be fully anonymized.

Important: If there are ethical or legal restrictions to sharing your data publicly, please explain these restrictions in detail. Please see our guidelines for more information on what we consider unacceptable restrictions to publicly sharing data: [http://journals.plos.org/plosone/s/data-availability#loc-unacceptable-data-access-restrictions](https://urldefense.com/v3/__http:/journals.plos.org/plosone/s/data-availability*loc-unacceptable-data-access-restrictions__;Iw!!LQC6Cpwp!rFVHO60ZNWeVXmKrYxR6pcOSVvXfm9Xixl7jUFVMroxX7drY1cdUKYzx3AzcnTfnP9xrQFDrb0GZpYHODw$). Note that it is not acceptable for the authors to be the sole named individuals responsible for ensuring data access.

We will update your Data Availability statement to reflect the information you provide in your cover letter.

[Note: HTML markup is below. Please do not edit.]

Reviewers' comments:

Reviewer's Responses to Questions

**Comments to the Author**

1. Is the manuscript technically sound, and do the data support the conclusions?

The manuscript must describe a technically sound piece of scientific research with data that supports the conclusions. Experiments must have been conducted rigorously, with appropriate controls, replication, and sample sizes. The conclusions must be drawn appropriately based on the data presented.

Reviewer #1: Yes

Reviewer #2: Yes

2. Has the statistical analysis been performed appropriately and rigorously?

Reviewer #1: N/A

Reviewer #2: N/A

3. Have the authors made all data underlying the findings in their manuscript fully available?

The [PLOS Data policy](https://urldefense.com/v3/__http:/www.plosone.org/static/policies.action*sharing__;Iw!!LQC6Cpwp!rFVHO60ZNWeVXmKrYxR6pcOSVvXfm9Xixl7jUFVMroxX7drY1cdUKYzx3AzcnTfnP9xrQFDrb0H1Hjt99Q$) requires authors to make all data underlying the findings described in their manuscript fully available without restriction, with rare exception (please refer to the Data Availability Statement in the manuscript PDF file). The data should be provided as part of the manuscript or its supporting information, or deposited to a public repository. For example, in addition to summary statistics, the data points behind means, medians and variance measures should be available. If there are restrictions on publicly sharing data—e.g. participant privacy or use of data from a third party—those must be specified.

Reviewer #1: Yes

Reviewer #2: Yes

4. Is the manuscript presented in an intelligible fashion and written in standard English?

PLOS ONE does not copyedit accepted manuscripts, so the language in submitted articles must be clear, correct, and unambiguous. Any typographical or grammatical errors should be corrected at revision, so please note any specific errors here.

Reviewer #1: Yes

Reviewer #2: Yes

5. Review Comments to the Author

Please use the space provided to explain your answers to the questions above. You may also include additional comments for the author, including concerns about dual publication, research ethics, or publication ethics. (Please upload your review as an attachment if it exceeds 20,000 characters)

Reviewer #1: The article concerns GPU-supported 3D simulations of the multicellular dynamics. Such simulations are very desired in many domains like medicine, biology, chemistry, etc. However, they are very time consuming. There were many attempts to speed up such simulations using different platforms: computer clusters, GPUs, etc. The authors have referenced them in the introduction section. In comparison to them, in this manuscript, a new GPU-based simulator is proposed (probably faster and at least as accurate as previous ones).

The solution is interesting and seems to be fast as well as provides accurate results. It is well described from the mathematical and biological perspective. However, the title and strength of the solution is ‘GPU-powered…”. Thus, the introduced improvements/parallelization should be described in more detail, e.g., figures and maybe pseudocode. Without this, the reader would have to scan the source code.

Below the summary of the remarks:

1. The solution needs more description from the GPU-parallelization perspective.

In the implementation details section we added a diagram (new figure 1) to better illustrate the overall program design and included more details about the parallization details of each module subsequent sections.

2. What was the CUDA version?

We used Cuda 11.2. We added the cuda version information inn the implementation deatils section.

3. There is no information about Gell simulations on CPU? time comparison with at least one-threaded version would be desired.

Gell, as the name suggests, does not have a CPU version due to the intrinsic limitations of CPU for large-scale cell simulation. An unoptimized CPU simulation code would not have provided an informative comparison. On the other hand, the well-cited and validated PhysiCell as a CPU simulator constitutes a more meaningful reference point for Gell. PhysiCell has been actively maintained by the developers and continuously optimized for multithread CPU. The speed gain against such an optimized and widely used CPU platform is more compelling than our ad hoc in-house implementation of CPU simulation. Moreover, we carefully constructed the comparison using the same cell model, reaction-diffusion solving algorithm, and the computational workload for both PhysiCell and Gell.

4. There is no information about GPU settings? And no information about their influence on the time and memory performance, what kind of memory does the GPU code use (shared, etc.)?

The optimal GPU settings are hardware dependent. We experimentally determined the optimal block size settings for our specific hardware configuration, which have been fixed for all simulations. Specifically, the kernels used for updating cell state had a 1D block width of 64, while the 3D kernel for oxygen consumption employed a block width of 4. The 2D kernels used for LOD calculation adopted a small 2D block width of 4 due to the storage requirements of the Thomas algorithm on GPU. This algorithm necessitates the storage of a temporary array for each thread, and a small block size allows us to store all data on the registers.

5. More GPU-support profiling would be desired.

The main profiling information is listed in Table 5.

6. Where are the conclusions?

A conclusion section was added.

7. Concerning Fig.8 and this case study: How the memory consumption was changed?

All the memory is allocated before the start of the simulation according to the max cell number and voxel number settings. The relationship between memory consumption with respect to max cell number and voxel number is linear, as listed in Table 4. We added another plot in Figure 9 (previously Figure 8) to illustrate that.

8. How the measurements in Table 5 were collected?

We used Nvidia Visual Profiler.

9. The authors have compared the proposed solution directly with one of CPU-parallelized simulators, why not with one of the previous GPU-supported ones?

The comparison method must meet three requirements: 1. It is open-source. 2. It solves the identical cell proliferation and interaction problem, and 3. The solved problems have been published and can be benchmarked against. PhysiCell is the only platform that meets these requirements. Other GPU solvers are either close-sourced or assume a different cell simulation model. Based on published information, these GPU solvers still suffer from computational limitations such as O(N^2) computational complexity and large memory footprint for relatively small problems. These limitations are summarized in the introduction as follows:

“Ya||a[18] is a paralleled agent-based model on GPU. Its extended spheroid cell model with spin-like polarities can simulate epithelial sheets and tissue polarity. Although Ya||a[18] can achieve 10X acceleration compared to CPU-based cell-based simulation library Chaste[19], their simulation software is not designed for large systems. Simulation of large systems using Ya||a[18] is limited by its computational complexity of O(N2) for cell-cell interaction. CBMOS[20] is another GPU-based software that provides a platform to study the effects of force functions, ODE solvers, time step sizes, and cellular events in CBMs. CBMOS[20] utilizes fast GPU vector operations provided by CuPy for efficient calculations and achieves a simulation speed 30X faster than their CPU version. Their emphasis, however, is on a better user interface for fast prototyping of new models. Its ability to handle large systems is still limited by the platform design, e.g., the force calculation time complexity is O(N2) and the GPU memory consumption can exceed 16 GB (e.g., NVIDIA Tesla T4) for >104 cells. GPU BioDynaMo[21] upgrades BioDynaMo[22] by enabling GPU co-processing. With a GTX 1080Ti GPU, GPU BioDynaMo can be 130X faster than the single thread CPU only version BioDynaMo. However, GPU BioDynaMo does not solve PDEs on GPU, and its uniform grid method for force calculation still needs CPU for linked list maintenance, which significantly limits its real-world performance.”

10. Equation 1, what does r_a means ?

r_a Is the transition rate of the stochastic transition a. We clarified this in the revised manuscript.

Reviewer #2: In this work the authors propose Gell, a GPU based approach to simulate large-scale systems on a cellular level. The results prove Gell efficiency in terms of speed-up, paving the way to novel and more in-depth analyses.
The paper is written in a good English and I enjoyed reading it.
However, there are many minors that need to be addressed before publication and one personal concern that I would like to see explicitly stated in the paper.

In the abstract the authors say "a fast and memory-efficient open-source GPU-based hybrid computational modeling platform Gell", I honestly had to read it a couple of times to understand that Gell was the name of the platform. I suggest them to "reverse" the sentence in something like "we develop Gell (GPU Cell), a fast and memory-efficient ...".

We rephrased that sentence as advised.

I think that this is a problem which will be addressed during the proof-reading process. Therefore I do not think the authors should worry about it now, but I prefer highlighting this problem: the bibliographic references are indicated with round brackets and not with squared brackets when squared brackets are usually used for the references.

We changed round brackets to squared brackets.

In line 86, the authors use the term "cluster computers", should not "computer cluster" be a better term?

We corrected that expression.

In line 89, I don't think that "shown" is the proper verb tense for the sentence. Also in line 94 the verb tense of "runs" should not be the proper one.

We corrected that expression.

Table 1 is not referenced in the text.

Table 1 is referenced as follows

The respective transition rates are listed in Table 1.

In line 190, it is not clear what is the v_i with the dot above, the authors should mention it during the explaination of the equation symbols.

v ̇_i is the acceleration. We added the explanation.

Moreover, the authors, should terminate each Equation with a dot or a comma and start the following sentence accordingly.

We corrected that issue.

In line 194, the authors state "is like fluid drag", I suggest them to use another term that is not "is like".

The description has been changed to “ηi is a fluid-drag-coefficient parameter”

In line 229, a column is missing to properly introduce the equation.

We are unsure about what column the reviewer was referring to. Here, the equation for any diffusive substance in the extracellular fluid domain Ω can be written as[24]

$$\begin{aligned} \frac{\partial\rho}{\partial t}=\nabla\cdot\left( D\nabla\rho\right)-\lambda\rho+S\left( \rho^{*}-\rho\right)-U\rho.\#\left( 11 \right) \end{aligned}$$

Depending on the problem, the domain boundary ∂Ω can be either Dirichlet or Neumann type. ρ is the substance concentration, ρ* is the saturation concentration, D is the diffusion coefficient, λ is the decay rate, S is the supply rate, U is the uptake rate.

In lines 260 and 261, a reference to the Thomes algorithm is required.

We added a cition of the following literature:

Povitsky A, Morris PJ. A Higher-Order Compact Method in Space and Time Based on Parallel Implementation of the Thomas Algorithm. J Comput Phys. 2000 Jun 10;161(1):182–203.

I suggest the authors to extend and improve the overall quality of the "Cell cycle update" Section, since it is very short and I have the feeling that some potentially interesting technical details are not fully explained.

We included more details about how the cell cycle update works in that section, as pasted below:

“The cell cycle module is responsible for managing cell proliferation, growth, and death. It is divided into two components, the cell cycle kernel and the cell birth-death submodule. The cell growth and phase transition only involve the current cell status as well as the local environmental factors. These processes are highly parallelizable and are directly distributed across different threads and computed by GPU in the Cell Cycle Kernel. The Cell Birth-Death Submodule manages cell proliferation and death, focusing on memory management of structured data. By leveraging this submodule, fast simulations can be conducted with only minimal additional computation and memory cost for thread competition avoidance. During cell proliferation, the total cell count is atomically updated to enable the addition of new daughter cells to the cell array. For cell death, cells earmarked for removal are initially labeled in their respective phase update thread, and then collectively deleted from the cell array in a subsequent kernel for efficient cell array maintenance. The overall computational complexity of the above-mentioned processes is approximately O(N).”

Figure 1 is not referenced in the text.

Figure 2 (previously Figure 1) is now referenced as follows

Figure 2 illustrates the voxel sorting method in a 2D scene.

In line 356 and following, I suggest the authors to avoid use and introduce acronyms in the sub-section titles and introduce them in the sub-section text.

We revised the sub-section title and removed the acronym. The new title is now simply “Hanging drop spheroid”

In line 364 a $\mu$m should be used instead of um, if I properly understood the unit of measure.
Also in line 393,438 there is the same problem in terms of unit of measure, I suggest the authors to check all the unit of measure notations and fix them accordingly (such as also in lines 465 and 466).

We revised according to the suggerstion.

I would also like to suggest the authors to use the explicit term to refer to Tables and Figures and not simply using "Tab" and "Fig".

We revised according to the suggerstion.

The specific of the personal computer listed in line 271 should be moved at the beginning of the Results Section (line 355).

We moved the spects to the Results section.

In line 416, "in vitro" should be attached as "in-vitro" and it should be in italic, since it is a latinism.

We corrected the word.

In Figure 6.c I would clearly point out that the x-axis is in logarithmic scale.

The logarithmic scale is pointed out. The new figure caption is:

“Gell simulation speedup with respect to PhysiCell with varying cell numbers (a), domain size voxel numbers (b), and PhysiCell CPU thread numbers (c with logarithmic x scale).”

In Figures 6.a and 6.b, I suggest the authors to reduce and move the x10^6 from the x label towards a more "tick-level" location.

Figure adjusted to million cells and million voxels.

In line 455, the acronym is used in the title: as suggested before I kindly ask to move the acronym inside the section text. Moreover there is a typo in "carcinima" which should be spelled in "carcinoma".

Corrected as suggested.

In lines 481 and 482, I think that the Table reference is wrong, shouldn't it be to Table 5?

Corrected as suggested.

In line 520, also the "in vivo" term should be in italic.

Corrected as suggested.

I renew for the x label of Figure 8 what I suggested before for Figure 6.

Corrected as suggested. Now x axis are labeled Million Cells instead of x10^6 Cells.

In line 553, the term "the" does not properly fit in my opinion, I suggest the authors to use "these" or something similar.

Corrected as suggested.

The only concern I have about this work is relative to the mathematical stability of Gell. In the work, the authors compare Gell with the state-of-the-art in terms of execution speed and speed-up, but they never claim or prove that the output of both models are the same. My concern is raised from the fact that typically GPUs work on a 32-bit precision while floating numbers on CPUs work on a 64-bit precision level. I would kindly ask the authors if they can check that the simulations performed for both the softwares (i.e., the simulations used to compute the speed-up) produce the same output. The authors could explicitly state that the results are the same, if this is actually the case as I expect.

Yes, we have compared the simulation output of Gell and PhysiCell on the HDS task using identical simulation parameters. Two simulations result in an identical spheroid radius of 1.87 mm and the same inner structures, as shown in Figure 3b. We note that there can be numerical differences due to round-off, random number generator, and parallelization settings, but the differences were negligible. Thus, we believe that Gell leads to stable and correct solutions.

As for concerns about using single precision variables, our observation is that the hybrid cell-based simulation does not require too many digits, and the solvers are very stable using the standard time step across the simulation. Additionally, while double-precision arithmetic can provide greater accuracy in certain calculations, simulations that involve biological systems are often subject to other sources of uncertainty, such as measurement error, variability in experimental conditions, and modeling approximations. Therefore, the additional precision provided by double-precision arithmetic may not necessarily lead to more accurate or reliable results in these cases.

6. PLOS authors have the option to publish the peer review history of their article ([what does this mean?](https://urldefense.com/v3/__https:/journals.plos.org/plosone/s/editorial-and-peer-review-process*loc-peer-review-history__;Iw!!LQC6Cpwp!rFVHO60ZNWeVXmKrYxR6pcOSVvXfm9Xixl7jUFVMroxX7drY1cdUKYzx3AzcnTfnP9xrQFDrb0Ft5JUccQ$)). If published, this will include your full peer review and any attached files.


If you choose “no”, your identity will remain anonymous but your review may still be made public.


**Do you want your identity to be public for this peer review?** For information about this choice, including consent withdrawal, please see our [Privacy Policy](https://urldefense.com/v3/__https:/www.plos.org/privacy-policy__;!!LQC6Cpwp!rFVHO60ZNWeVXmKrYxR6pcOSVvXfm9Xixl7jUFVMroxX7drY1cdUKYzx3AzcnTfnP9xrQFDrb0E0j5Vb5w$).

Reviewer #1: No

Reviewer #2: **Yes:** Daniele Maria Papetti

[NOTE: If reviewer comments were submitted as an attachment file, they will be attached to this email and accessible via the submission site. Please log into your account, locate the manuscript record, and check for the action link "View Attachments". If this link does not appear, there are no attachment files.]

While revising your submission, please upload your figure files to the Preflight Analysis and Conversion Engine (PACE) digital diagnostic tool, [https://pacev2.apexcovantage.com/](https://urldefense.com/v3/__https:/pacev2.apexcovantage.com/__;!!LQC6Cpwp!rFVHO60ZNWeVXmKrYxR6pcOSVvXfm9Xixl7jUFVMroxX7drY1cdUKYzx3AzcnTfnP9xrQFDrb0GjTCs3fA$). PACE helps ensure that figures meet PLOS requirements. To use PACE, you must first register as a user. Registration is free. Then, login and navigate to the UPLOAD tab, where you will find detailed instructions on how to use the tool. If you encounter any issues or have any questions when using PACE, please email PLOS at [figures@plos.org](mailto:figures@plos.org). Please note that Supporting Information files do not need this step.

Response to Reviewers
